# Impaired antigen clearance of macrophages associates with heart failure and non-obstructive azoospermia: insights from transcriptomics, in silico knockout, and *in vitro* validation

**DOI:** 10.3389/fimmu.2026.1909437

**Published:** 2026-09-15

**Authors:** Zheng Zhao, Rong Li, Kai Li, Rui Zhang, Jiawei Tian, Da Teng, Xiaoyan Zhou, Baoqi Jia, Xianmiao Yuan, Hui Xin

**Affiliations:** 1 Department of Cardiology, The Affiliated Hospital of Qingdao University, Qingdao University, Qingdao, China; 2 Department of Cardiology I, Qingdao Central Hospital, University of Health and Rehabilitation Sciences, Qingdao, China; 3 Department of Cardiology, Qingdao Hospital, University of Health and Rehabilitation Sciences (Qingdao Municipal Hospital), Qingdao, China

**Keywords:** heart failure (HF), non-obstructive azoospermia (NOA), antigen clearance, macrophages, in silico knockout

## Abstract

**Background:**

Heart failure (HF) and non-obstructive azoospermia (NOA) are high-burden diseases that share chronic inflammation and tissue injury. Macrophages are key regulators in both conditions, yet whether a common molecular basis exists remains unclear.

**Methods:**

We analyzed HF and NOA expression profiles from the GEO database using WGCNA and machine learning to screen for core shared genes. Diagnostic performance was evaluated using AUC in independent validation sets. Single-cell RNA-seq mapped the cellular origin of core genes. Immune infiltration, pathway activity, and transcription factor networks were analyzed using xCell, PROGENy, and DoRothEA. To investigate functional roles, we applied single-cell in silico knockout to macrophages and designed *in vitro* knockdown experiments for validation.

**Results:**

*SLAMF7*, *IL2RA*, and *PRF1* were identified as core shared genes. The diagnostic model achieved AUCs of 0.782 (HF) and 0.724 (NOA) in validation sets. All three genes were highly expressed in macrophages, and their mean expression correlated with M2-like macrophages as well as *JAK-STAT* and *NF-κB* pathway activities. Transcription factor network analysis revealed *NFKB1*, *STAT1*, *STAT3*, *STAT4* and *STAT5A/B* as upstream regulators. Any gene loss upregulated C1q, scavenger receptors, the NLRP3 inflammasome, and inflammatory factors. Distinct context-specific changes were observed: in NOA macrophages, *PRF1* loss caused ectopic expression of sperm protamine genes (*PRM2*, *TNP1*); *IL2RA* loss activated the *IRF8–TLR2* axis; and *SLAMF7* loss enhanced MHC-II and interferon pathways. In HF macrophages, *SLAMF7* loss activated the prostaglandin pathway; *IL2RA* loss induced upregulation of M2-like markers *FOLR2* and *LYVE1*; and *PRF1* loss enhanced C1q expression. *In vitro* knockdown experiments showed that, without antigen stimulation, knockdown of all three genes upregulated *C1qa*, *Folr2*, *Il1b*, *Acod1* and *Nlrp3*. Under sperm stimulation, *PRF1* knockdown increased *Prm2* and *S100a8*, whereas *IL2RA* knockdown increased *Irf8*. Under cardiac lysate stimulation, *PRF1* knockdown led to ectopic expression of the cardiac-specific genes *Myh6* and *Tnnt2*.

**Conclusion:**

*SLAMF7*, *IL2RA* and *PRF1* form a negative immune regulatory hub in macrophages that links HF and NOA. Loss of these genes is associated with a conserved complement–inflammation module, whereas the disease outcome is determined by organ-specific antigens. Targeting this shared hub may offer a potential therapeutic direction for future investigation.

## 1 Introduction

Heart failure (HF) represents the end stage of various cardiac diseases, characterized by reduced ventricular function and pulmonary or systemic congestion (1, 2). Non-obstructive azoospermia (NOA) is the most severe form of male infertility, defined by spermatogenic failure in the testis (3) and involving immune dysregulation, infectious factors, and abnormalities in spermatogenesis-related genes (4–8). Despite affecting different organs, these two diseases share chronic sterile inflammation and tissue injury as core pathological features (9, 10). HF patients exhibit elevated circulating levels of pro-inflammatory cytokines, accompanied by macrophage and T cell infiltration in the myocardium (11–13). Similarly, NOA patients display lymphocyte infiltration in testicular tissue along with the production of anti-sperm antibodies (14, 15).

Macrophages, as core cells of innate immunity, play critical roles in tissue homeostasis, injury repair, and inflammatory regulation (16). In HF, cardiac macrophages are responsible for clearing necrotic cardiomyocytes, regulating inflammatory responses, and modulating fibrosis (17, 18). In NOA, testicular macrophages participate in the phagocytosis of apoptotic sperm and the maintenance of the immune-privileged microenvironment (19, 20). Although macrophages in these two tissues perform similar functions, they reside in entirely distinct tissue microenvironments. Whether macrophages from different organs share a common molecular regulatory program remains unknown. If such a program exists, its dysregulation may produce distinct disease phenotypes depending on the tissue context. We therefore hypothesize that certain negative immune regulatory genes are commonly inactivated in both diseases, driving macrophages into a conserved inflammatory state, while the type of tissue-specific antigen ultimately determines whether HF or NOA develops.

To test this hypothesis, we performed a systematic analysis of publicly transcriptomic data to screen for core shared genes between HF and NOA. The analysis integrated weighted gene co-expression network analysis (WGCNA), machine learning, single-cell analysis, immune infiltration assessment, pathway activity evaluation, and transcription factor regulatory network analysis. We further conducted in silico knockout simulations to reveal the common and cell-type-specific effects of gene deficiency, and designed *in vitro* knockdown experiments to validate the key findings. This study aimed to establish a mechanistic model of shared immunopathology between these two diseases and to provide a theoretical foundation for developing diagnostic and therapeutic strategies targeting both conditions.

## 2 Materials and methods

### 2.1 Data acquisition and preprocessing

The HF-related datasets GSE5406 and GSE57338, and the NOA-related datasets GSE45885 and GSE45887, were downloaded from the Gene Expression Omnibus (GEO) repository (https://www.ncbi.nlm.nih.gov/geo/). GSE57338 is a microarray subdataset of the SuperSeries GSE57345, generated on the GPL11532 platform (Affymetrix Human Gene 1.1 ST Array). The RNA-seq subdataset within this SuperSeries (GSE57344, platform GPL9052) was not included in this study. Raw CEL files were processed with the affy package and RMA normalization. Probe annotation was performed using the hugene10sttranscriptcluster.db package, with Entrez IDs as gene identifiers. The ComBat algorithm was then applied separately to merge the HF and NOA training sets to remove batch effects, generating the normalized expression matrices HF_combat and NOA_combat. The external validation set for HF was GSE57338, and for NOA was GSE25518. Single-cell data for HF hearts were obtained from GSE183852, and for NOA testes from GSE149512. Bioinformatics analyses were performed in accordance with the MIAME guidelines for microarray data reporting.

### 2.2 Weighted gene co-expression network analysis

We performed WGCNA on HF_combat and NOA_combat. After selecting a soft thresholding power to achieve a scale-free topology, we identified co-expression modules using the dynamic tree-cutting algorithm. We then calculated module-trait correlations and selected modules with positive correlation (cor > 0) and p < 0.05. Finally, we performed Gene Ontology (GO) enrichment analysis on each positively correlated module using clusterProfiler (p.adjust < 0.05) and retained modules enriched for immune-related terms.

### 2.3 Candidate gene intersection and machine learning feature selection

The intersection of genes from the immune-related positively correlated modules of HF and NOA was taken as a set of common candidates. This set was mapped to the STRING protein-protein interaction (PPI) network, and the MCODE plugin was used to extract core clusters. Meanwhile, multiple centrality measures (Betweenness, Closeness, Degree, Eigenvector, Information, LAC, Network, and Subgraph) were calculated using CytoNCA, and genes significant in all eight measures were selected. The union of the MCODE and CytoNCA results formed the final candidate gene set. Five machine learning algorithms (LASSO regression, Elastic Net, Random Forest, XGBoost, and Ridge regression) were then applied for feature selection. Each algorithm was run with five-fold cross-validation, and the selection frequency of each gene was recorded. Genes selected by at least four algorithms in both diseases were designated as core shared genes.

### 2.4 Diagnostic model construction and evaluation

A logistic regression model was constructed using the expression levels of the core genes. Receiver Operating Characteristic (ROC) curves and the area under the curve (AUC) were calculated for the training and validation sets to evaluate the discriminative ability of the model.

### 2.5 Single-cell transcriptome analysis

Seurat v4 was used for quality control, normalization (LogNormalize), principal component analysis, Uniform Manifold Approximation and Projection (UMAP) dimensionality reduction, and clustering of the HF and NOA single-cell objects. Cell types were annotated based on known marker genes. The expression levels of the core genes were then extracted, and their distribution across cell subpopulations was analyzed.

### 2.6 Immune infiltration and pathway activity analysis

Enrichment scores for 64 immune cell types were estimated using xCell from bulk transcriptomic data. For each sample, a composite score defined as the average expression of the core genes was correlated with immune cell abundances and PROGENy pathway scores using Spearman analysis with FDR correction.

### 2.7 Transcription factor regulatory network analysis

Human transcription factor-target gene relationships were downloaded from the DoRothEA database. Transcription factors directly regulating the core genes were screened, and all of their target genes were extracted. GO biological process enrichment analysis was then performed using clusterProfiler with an adjusted p-value cutoff of 0.05 and a minimum gene set size of 5 to reveal the functional tendencies of these target genes.

### 2.8 In silico knockout simulation

Macrophage subpopulations were extracted from the HF and NOA single-cell Seurat objects. For each subpopulation, highly variable genes were first calculated using FindVariableFeatures with nfeatures = 2000, and the target gene was forcibly added to the variable gene list. Cell types with more than 500 cells were randomly downsampled to 500 cells. The raw count matrix was then extracted, retaining only the variable genes and the target gene. Gene regulatory networks were constructed using the scTenifoldKnk package with the knockout method, where the target gene served as the knockout gene. The number of random networks was set to 5, and the number of cells sampled per network was defined as the minimum of 500 and the actual cell count, with other parameters kept as default. By comparing the original and knockout networks, the distance, fold change (FC), and adjusted p-value (p.adj) were calculated for each gene. Genes with p.adj < 0.05 and an absolute log_2_ fold change greater than 1 were considered significantly differentially expressed.

### 2.9 *In vitro* macrophage functional validation

RAW 264.7 cells, HL-1 cells, and TM4 cells were obtained from a collaborating laboratory at Qingdao Medical College of Qingdao University, where they were originally sourced from commercial suppliers. According to local legislation and institutional requirements, ethical approval was not required for the use of these commercially derived, deidentified cell lines. Cells were divided into four groups: siNC, si*IL2RA*, si*PRF1*, and si*SLAMF7*, and transfected with siRNA at 20 nM for 48 hours. Under no antigen stimulation, Quantitative Real-Time PCR (qPCR) measured mRNA levels of *C1qa*, *Folr2*, *Il1b*, *Acod1*, *Hpgds*, *Ccl5* and *Nlrp3*, and Enzyme-Linked Immunosorbent Assay (ELISA) determined prostaglandin D2 (PGD2) concentration in the supernatant.

For NOA-like conditions, sperm were collected from the epididymis of healthy male C57BL/6 mice (HFK Bioscience Co., Ltd., Beijing, China). All animal procedures were approved by the Animal Care and Use Committee of The Affiliated Hospital of Qingdao University and were performed in accordance with the Regulations for the Administration of Laboratory Animals. After three freeze-thaw cycles and UV inactivation, sperm were added to transfected macrophages at 1×10^5^/mL. Following 4 hours of co-culture, extracellular sperm were washed away and incubation continued for 24 hours, after which qPCR measured *Prm2*, *Irf8*, and *S100a8*. For HF-like conditions, HL-1 cells were subjected to three freeze-thaw cycles and centrifuged to obtain necrotic cardiomyocyte lysate (50 μg/mL), followed by qPCR detection of *Myh6*, *Tnnt2*, *Folr2*, *Hpgds*, and *C1qa* under the same treatment procedure.

Additionally, to assess whether macrophage-derived soluble factors could affect target cells, culture supernatants were collected from each group 48 h post-transfection. After centrifugation and filtration, the supernatants were applied to HL-1 cells and TM4 cells as conditioned media for 24 h. mRNA levels of *Il6*, *Tnfa*, *Il1b*, and *Ccl2* in HL-1 cells, and *Il6*, *Tnfa*, *Il1b*, *Gata4*, *Wt1*, and *Tjp1* in TM4 cells, were then measured by qPCR. qPCR primer sequences are provided in **Supplementary Table S8**.

To further assess the degradation of internalized antigens, pHrodo-based flow cytometry was performed. Sperm or cardiomyocyte lysate was labeled with pHrodo and incubated with the corresponding macrophage groups. After 24 h, mean fluorescence intensity (MFI) of each group was measured by flow cytometry. Additionally, Western blot was performed to verify *PRF1* expression and its role in antigen degradation at the protein level. *PRF1* expression was examined in RAW 264.7 cells with or without lipopolysaccharide (LPS) plus interferon-gamma (IFN-γ) stimulation for 24 h, using lysis buffer alone as a blank control. For antigen degradation assays, cells were collected 24 h after treatment under the same conditions as those used in the qPCR experiments. Total protein was extracted, separated by sodium dodecyl sulfate-polyacrylamide gel electrophoresis (SDS-PAGE), and transferred to polyvinylidene difluoride (PVDF) membranes, followed by incubation with primary antibodies against *PRF1*, *PRM1*, *PRM2*, *MYH6*, and *TNNT2*, and horseradish peroxidase (HRP)-conjugated secondary antibodies. Signals were visualized by enhanced chemiluminescence (ECL) and quantified using ImageJ, with β-actin as the loading control.

### 2.10 Statistical analysis

All statistical analyses were performed using R version 4.2.0. The Wilcoxon rank-sum test was used for comparisons of continuous variables, and Spearman’s rank correlation coefficient was applied for correlation analysis. For multiple group comparisons, one-way analysis of variance (ANOVA) was conducted, followed by Tukey’s HSD post-hoc test for multiple comparison correction. *In vitro* experimental data are presented as mean ± standard deviation (SD). A p-value < 0.05 was considered statistically significant.

## 3 Results

### 3.1 Identification of immune-related WGCNA modules and shared candidate genes

WGCNA was performed separately on the HF and NOA datasets to identify immune-related genes shared by the two diseases (**Figures 1A, B**; **Supplementary Figure S1**). In the HF dataset, ME13 was positively correlated with disease status and enriched in immune terms including T cell differentiation and lymphocyte-mediated immunity (**Supplementary Table S1**). In the NOA dataset, ME1 was similarly positively correlated and enriched in TNF family cytokine production and inflammatory response regulation (**Supplementary Table S2**). The intersection of ME13 and ME1 yielded 35 shared immune-related candidate genes (**Supplementary Table S3**). Functional enrichment analysis of these genes revealed significant enrichment in GO biological processes including regulation of inflammatory response, cytokine production, leukocyte cell-cell adhesion, and lymphocyte activation (**Figure 1C**). Cellular component analysis primarily assigned them to the external side of the plasma membrane, the immunological synapse, and cell surface receptor complexes (**Figure 1D**). Molecular function analysis implicated tumor necrosis factor receptor binding and cytokine receptor activity (**Figure 1E**). KEGG pathway analysis further showed enrichment in immune-related pathways such as Th1/Th2/Th17 differentiation, cytokine-cytokine receptor interaction, and primary immunodeficiency (**Figure 1F**). Thus, these 35 genes constitute a core immune gene set linking HF and NOA, functioning in immune regulation, inflammation, and cell-cell communication.

**Figure 1:**
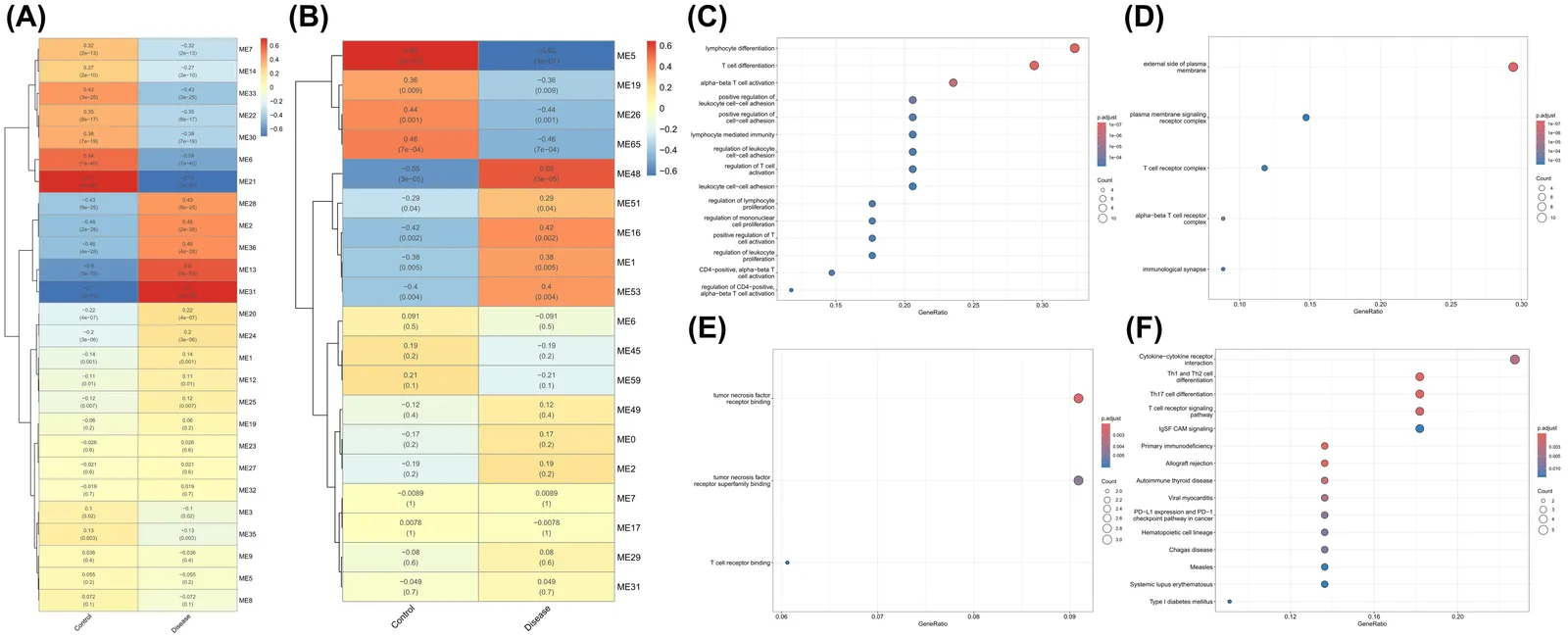
Screening and functional enrichment of common immune candidate genes. **(A)** Module-trait correlation heatmap for HF. **(B)** Module-trait correlation heatmap for NOA. **(C)** GO biological process enrichment. **(D)** GO cellular component enrichment. **(E)** GO molecular function enrichment. **(F)** KEGG pathway enrichment.

### 3.2 Identification and validation of core shared genes

A PPI network was constructed from the 35 common immune candidate genes (**Figure 2A**). The MCODE plugin extracted a core cluster containing 11 genes (**Figure 2B**), while CytoNCA identified 12 genes (**Figure 2C**). The union of the two methods yielded 12 candidate genes (**Figure 2D**). To further screen for the most robust cross-disease core genes from these 12 candidates, we applied five machine learning algorithms to both the HF and NOA training sets. The results showed that *PRF1* and *IL2RA* were selected by all five algorithms in both diseases, whereas *SLAMF7* was selected by five algorithms in HF and four in NOA, indicating substantially higher stability than the other candidates. Based on selection frequency and biological relevance, we ultimately identified *PRF1*, *IL2RA* and *SLAMF7* as the core shared genes (**Supplementary Table S4**). A logistic regression diagnostic model was then constructed based on the expression levels of these three genes. In the training sets, the model achieved AUCs of 0.731 for HF and 0.846 for NOA; in the independent validation sets, the AUCs were 0.782 and 0.724, respectively (**Figures 2E–H**).

**Figure 2:**
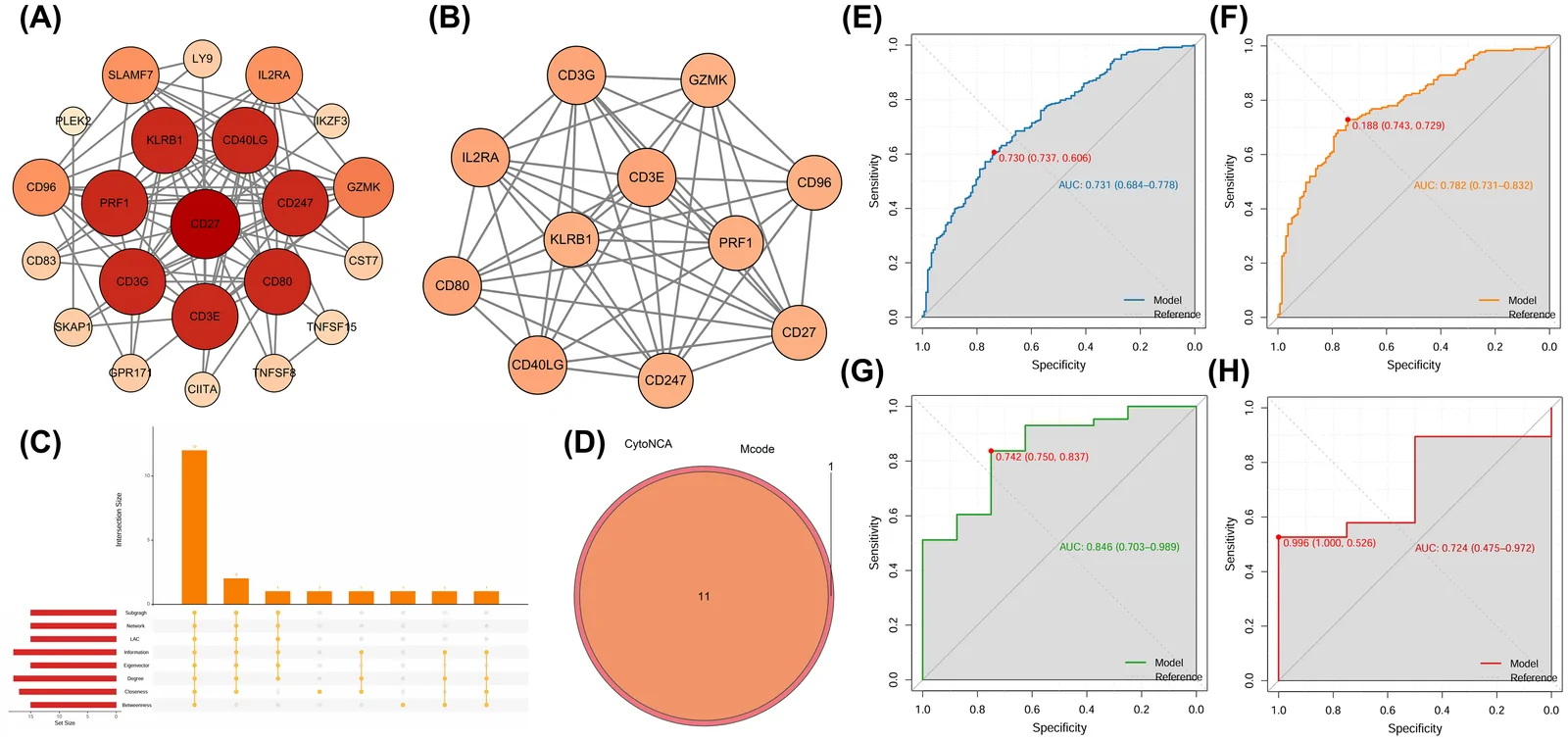
Core gene screening and diagnostic model validation. **(A)** PPI network. **(B)** MCODE core cluster. **(C)** UpSet plot of CytoNCA. **(D)** Venn diagram of MCODE and CytoNCA results. **(E)** ROC curve for the HF training set. **(F)** ROC curve for the HF validation set. **(G)** ROC curve for the NOA training set. **(H)** ROC curve for the NOA validation set.

### 3.3 Single-cell localization of core genes

The cellular origin of the three core genes was determined by analyzing single-cell transcriptomic data from HF cardiac tissue and NOA testicular tissue (**Figure 3**). The results showed that *SLAMF7*, *IL2RA*, and *PRF1* were clearly expressed in macrophages from both tissues (**Supplementary Tables S5**, **6**), suggesting that these genes may regulate macrophage function to influence disease processes. Although these genes were also present in other immune cells, macrophages are central to innate immunity, tissue homeostasis, and antigen clearance. Accordingly, we focused our subsequent mechanistic studies on macrophages.

**Figure 3:**
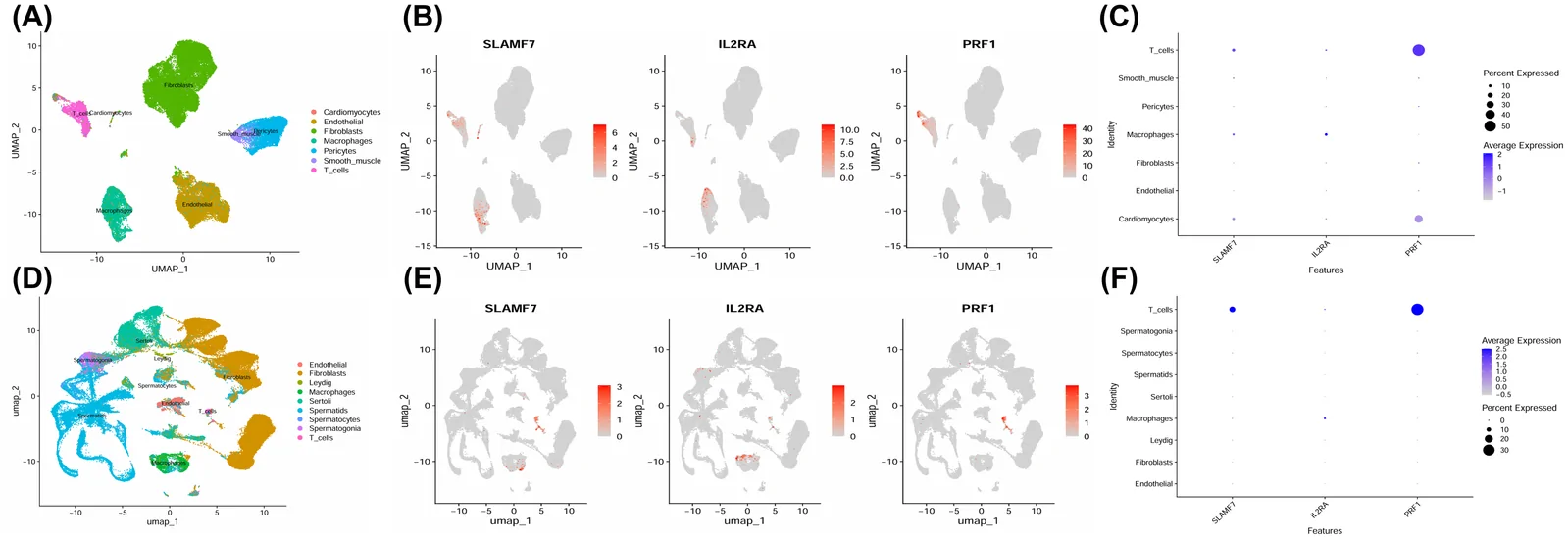
Single-cell expression localization of core genes in HF and NOA. **(A)** UMAP cell atlas of the HF heart. **(B)** UMAP expression plots of core genes in the HF heart. **(C)** Dot plot of the core genes in the HF heart. **(D)** UMAP cell atlas of the NOA testis. **(E)** UMAP expression plots of core genes in the NOA testis. **(F)** Dot plot of core genes in the NOA testis.

### 3.4 Correlation of the gene score with immune features

A composite score was calculated for each sample as the average expression of the three core genes. Spearman correlation analysis was then performed between this composite score and xCell-derived immune cell abundances, as well as PROGENy-derived pathway activities. Immune infiltration analysis revealed that, in both disease settings, the composite score was significantly positively correlated with macrophages, particularly the M2-like subtype. In addition, the composite score in NOA also showed positive correlations with M1 macrophages and total macrophages (**Figure 4A**). Pathway activity analysis indicated that, in NOA, the composite score was positively correlated with the *TGF-β*, *JAK-STAT*, and *NF-κB* pathways. In HF, the composite score was positively correlated with the *NF-κB* and *TNF-α* pathways (**Figure 4B**).

**Figure 4:**
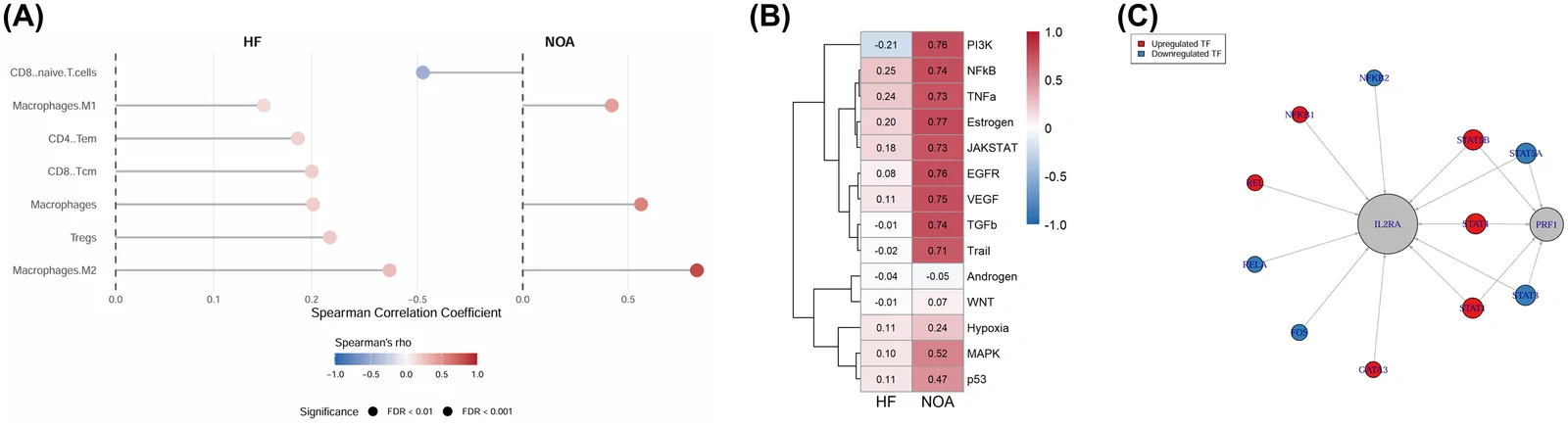
Association of core genes with immune microenvironment and transcription factor regulation. **(A)** Lollipop plot of immune infiltration correlations. **(B)** Heatmap of pathway activity correlations. **(C)** Transcription factor regulatory network.

### 3.5 Transcription factor regulatory network analysis of core genes

Using the DoRothEA database, we constructed a transcription factor–target gene regulatory network. This analysis revealed that *SLAMF7*, *IL2RA*, and *PRF1* are co-regulated by multiple immune-related transcription factors, including the *NF-κB* family (*NFKB1*, *RELA*), the *STAT* family (*STAT1*, *STAT3*, *STAT4*, *STAT5A/B*), *GATA3*, and *FOS* (**Figure 4C**). Detailed expression, activity, and correlation data for these transcription factors are provided in **Supplementary Table S7**. We then performed GO enrichment analysis on all target genes of these transcription factors. The results showed significant enrichment in terms related to T cell activation, lymphocyte proliferation, cytotoxicity, interferon response, and regulation of inflammatory response. These findings further support the central role of the three core genes in the shared immune-inflammatory pathology between HF and NOA.

### 3.6 In silico knockout analysis

To reveal functional changes in macrophages following loss of the three core genes, differential expression profiles of macrophages derived from HF and NOA models were analyzed after individual knockout of *IL2RA*, *PRF1*, or *SLAMF7* (**Figure 5**).

**Figure 5:**
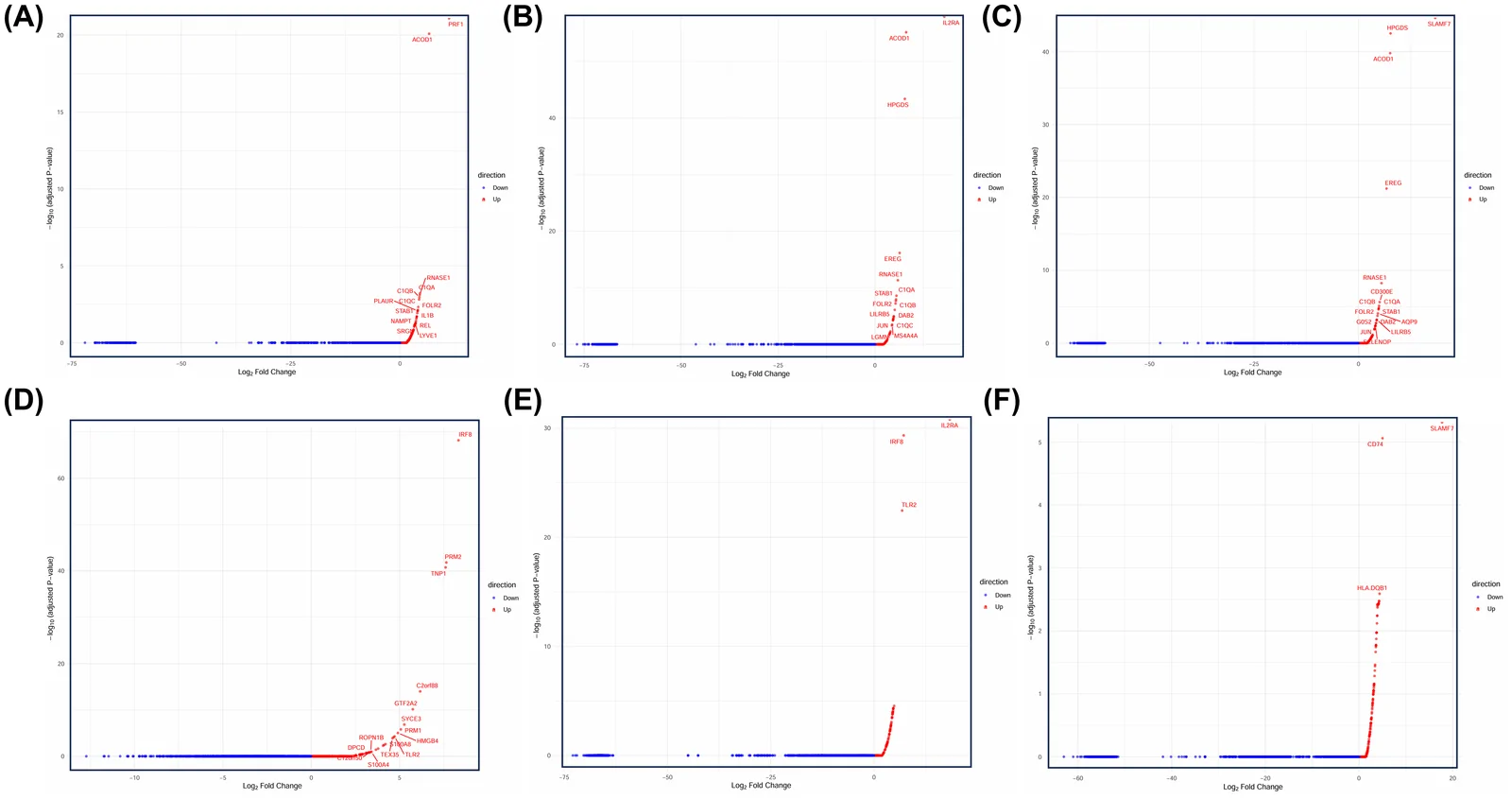
Differential expression profiles of macrophages after core gene knockout. **(A–C)** Volcano plots of *PRF1*, *IL2RA*, and *SLAMF7* knockout in HF macrophages. **(D–F)** Volcano plots of *PRF1*, *IL2RA*, and *SLAMF7* knockout in NOA macrophages.

#### 3.6.1 Common transcriptional response analysis

Knockout of any of the three genes, whether in HF or NOA, consistently upregulated a similar set of genes, including complement components *C1QA*, *C1QB* and *C1QC*, scavenger receptors *STAB1*, *FOLR2* and *MS4A4A*, inflammatory signaling molecules *NFKBIA* and *IL1B*, the metabolic regulator *ACOD1*, the chemokine *CCL5*, and the NLRP3 inflammasome. Thus, *IL2RA*, *PRF1*, and *SLAMF7* normally suppress complement activation, limit inflammation, and are associated with the expression of metabolism-related genes in macrophages across both diseases. Their loss leads to a conserved complement-inflammation module.

#### 3.6.2 Differential expression analysis under sperm stimulation

In NOA testicular macrophages, knockout of the three genes produced distinct effects on sperm-related gene expression. *PRF1* knockout strongly upregulated the protamine genes *PRM2*, *TNP1*, and *PRM1*, along with *IRF8*, *S100A8*, and *S100A9*. In contrast, *IL2RA* knockout upregulated *IRF8* and *TLR2* as well as many spermatogenesis-related genes (e.g., *HSPB9*, *SEPT12*), but did not affect protamine genes. *SLAMF7* knockout primarily enhanced major histocompatibility complex class II (MHC-II) molecules (e.g., *CD74*, *HLA-DQB1*) and interferon pathway genes, with no impact on sperm genes. These results indicate that *PRF1* is the core effector for degrading chromatin of phagocytosed sperm in macrophages. *IL2RA* mainly regulates the *IRF8–TLR2* axis and antigen presentation, while *SLAMF7* primarily suppresses the MHC-II-mediated antigen presentation pathway. Together, these three genes play complementary roles in maintaining immune privilege and clearance capacity in testicular macrophages. Loss of any one of them may disrupt normal processing of sperm antigens, thereby triggering anti-sperm immune responses, a critical step in NOA pathogenesis.

#### 3.6.3 Differential expression analysis under cardiac stimulation

In HF cardiac macrophages, knockout of the three genes produced distinct inflammatory profiles. *SLAMF7* knockout primarily activated the prostaglandin pathway and C1q, suggesting that *SLAMF7* negatively regulates prostaglandin-mediated inflammation. *IL2RA* knockout significantly upregulated the M2-like markers *FOLR2* and *LYVE1*, indicating that *IL2RA* may inhibit macrophage polarization toward a pro-fibrotic phenotype. *PRF1* knockout mainly enhanced C1q expression, and complement activation is a key step in sterile inflammation following myocardial injury. Together, these results reveal that the three genes cooperate through different mechanisms, including prostaglandin regulation, M2 polarization control, and complement suppression, to limit excessive macrophage inflammation in the cardiac microenvironment. In HF, self-antigens released from necrotic cardiomyocytes (e.g., myosin) require macrophage clearance. Given the conserved upregulation of complement and scavenger receptors observed in our knockout experiments, we propose that loss of any of the three genes may impair cardiac antigen clearance. Specifically, *PRF1* deficiency leads to complement-dependent inflammatory amplification, *IL2RA* deficiency may promote polarization toward fibrosis-associated macrophages, and *SLAMF7* deficiency exacerbates prostaglandin-mediated tissue damage. The combined loss of these functions collectively promotes chronic inflammation and ventricular remodeling in HF.

### 3.7 *In vitro* knockdown validation

Knockdown of *IL2RA*, *PRF1*, or *SLAMF7* in RAW 264.7 cells significantly increased the mRNA levels of *C1qa*, *Folr2*, *Il1b*, *Acod1*, and *Nlrp3* in the absence of antigen stimulation, with *IL2RA* knockdown showing the strongest induction of *Folr2*, *PRF1* knockdown most prominently upregulating *Il1b* and *Nlrp3*, and *SLAMF7* knockdown specifically increasing *Hpgds* expression and *PGD2* secretion (**Figures 6A–H**). Under antigen stimulation, *PRF1* knockdown led to significant accumulation of *Prm2* along with upregulation of *Irf8* and *S100a8* in the sperm co-culture model, whereas *IL2RA* knockdown only increased *Irf8* and *SLAMF7* knockdown had no significant effect (**Figures 6I–K**). Similarly, in the cardiomyocyte lysate model, *PRF1* knockdown induced ectopic expression of *Myh6* and *Tnnt2* and enhanced *C1qa* expression, while *IL2RA* knockdown selectively upregulated *Folr2* and *SLAMF7* knockdown activated *Hpgds* (**Figures 6L–P**). Conditioned media from each knockdown group applied to HL-1 cells and TM4 cells further showed that macrophage dysfunction could affect target cells via paracrine signaling, with *PRF1* knockdown producing the strongest effects across both cell types, whereas *SLAMF7* knockdown showed the greatest induction of *Ccl2*, consistent with their respective macrophage phenotypes (**Figure 7**).

**Figure 6:**
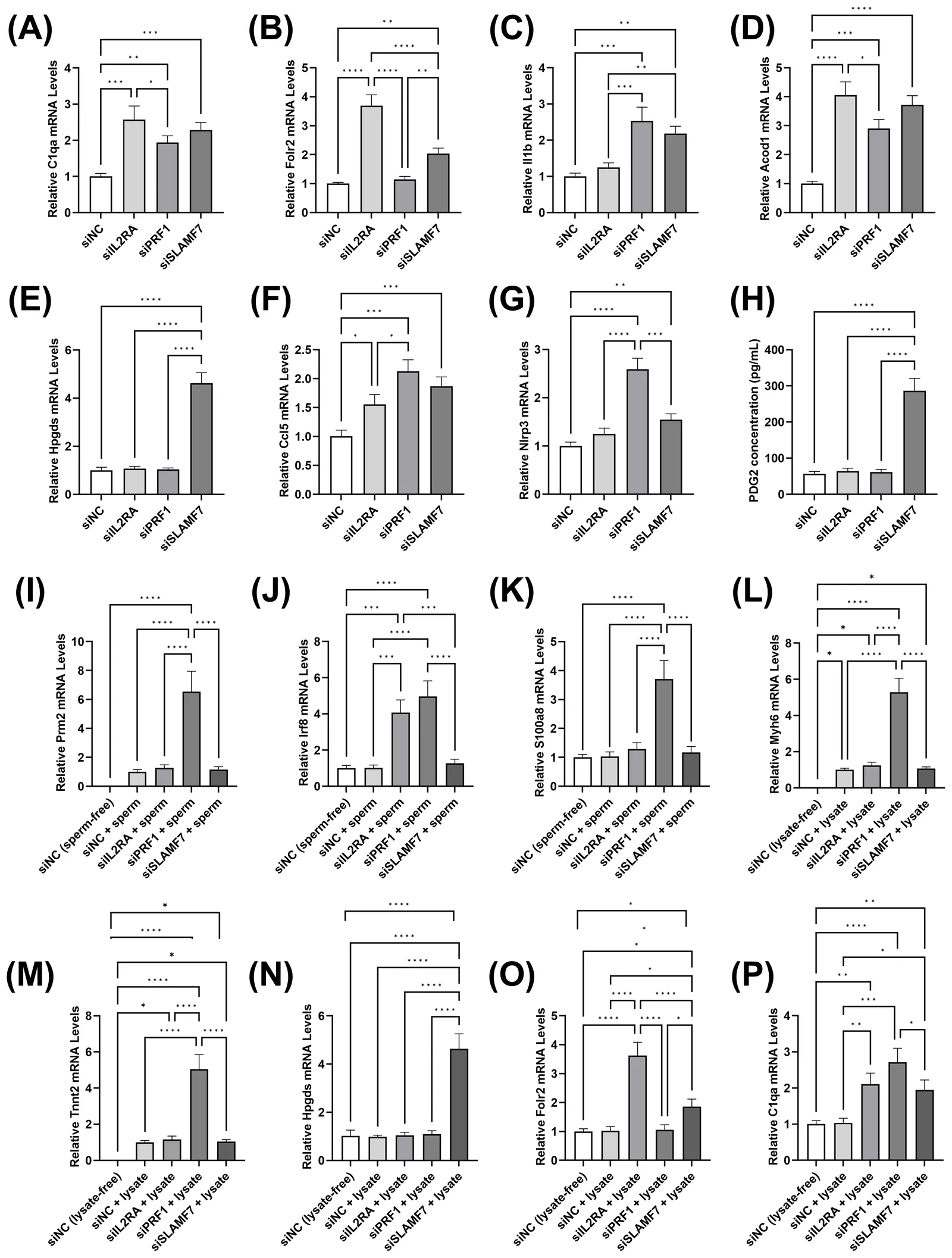
qPCR and ELISA validation of core gene functions in macrophages following *in vitro* knockdown. **(A–H)** mRNA levels of *C1qa*, *Folr2*, *Il1b*, *Acod1*, *Hpgds*, *Ccl5*, *Nlrp3* and supernatant PGD2 concentration in each knockdown group under no antigen stimulation. **(I–K)** mRNA levels of *Prm2*, *Irf8*, and *S100a8* in each treatment group under sperm co-culture. **(L–P)** mRNA levels of *Myh6*, *Tnnt2*, *Hpgds*, *Folr2*, and *C1qa* in each treatment group under cardiac lysate stimulation. Data are shown as mean ± SD (n = 3). *p < 0.05, **p < 0.01, ***p < 0.001, and ****p < 0.0001.

**Figure 7:**
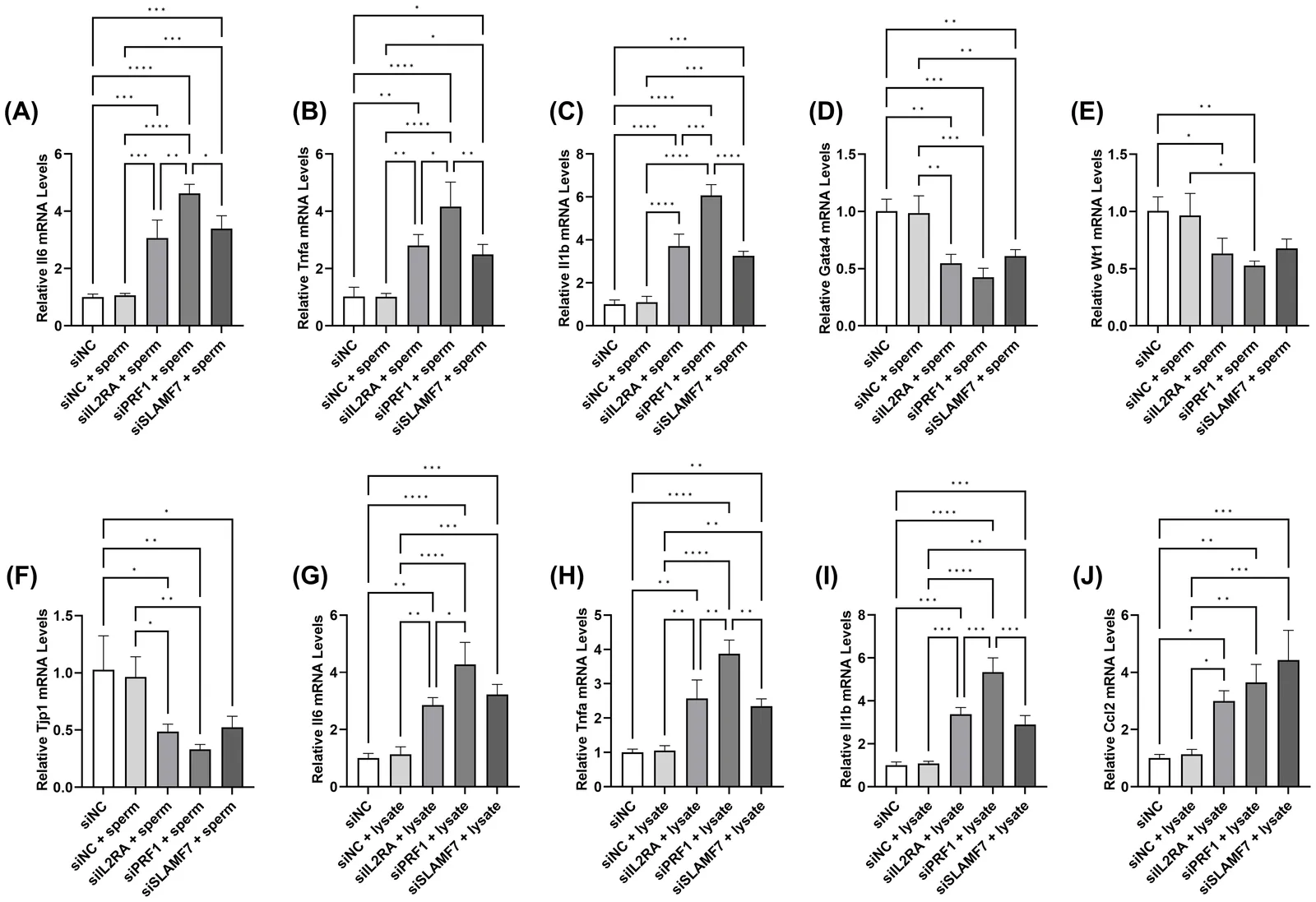
Conditioned medium experiments validating the effects of macrophage-derived soluble factors on target cells. **(A–F)** mRNA levels of *Il6*, *Tnfa*, *Il1b*, *Gata4*, *Wt1*, and *Tjp1* in TM4 cells; **(G–J)** mRNA levels of *Il6*, *Tnfa*, *Il1b*, and *Ccl2* in HL-1 cells. Data are shown as mean ± SD (n = 3). *p < 0.05, **p < 0.01, ***p < 0.001, and ****p < 0.0001.

We next assessed the degradation of internalized antigens using pHrodo-based flow cytometry. In both sperm and cardiomyocyte lysate treatment models, *PRF1* knockdown significantly increased pHrodo fluorescence intensity compared with controls, whereas *IL2RA* and *SLAMF7* knockdown resulted in only modest increases (**Supplementary Figure S2**). Consistent with these observations, Western blot showed that *PRF1* was barely detectable in resting macrophages but markedly upregulated upon LPS plus IFN-γ stimulation (**Figures 8A, B**). Following *PRF1* knockdown, *PRM1* and *PRM2* protein bands persisted at 24 h post-phagocytosis in the sperm model, and *MYH6* and *TNNT2* remnants were similarly elevated in the cardiomyocyte lysate model, whereas they were nearly undetectable in controls (**Figures 8C–H**). These results indicate that *PRF1* deficiency impairs antigen degradation at the protein level, corroborating the qPCR and pHrodo flow cytometry findings.

**Figure 8:**
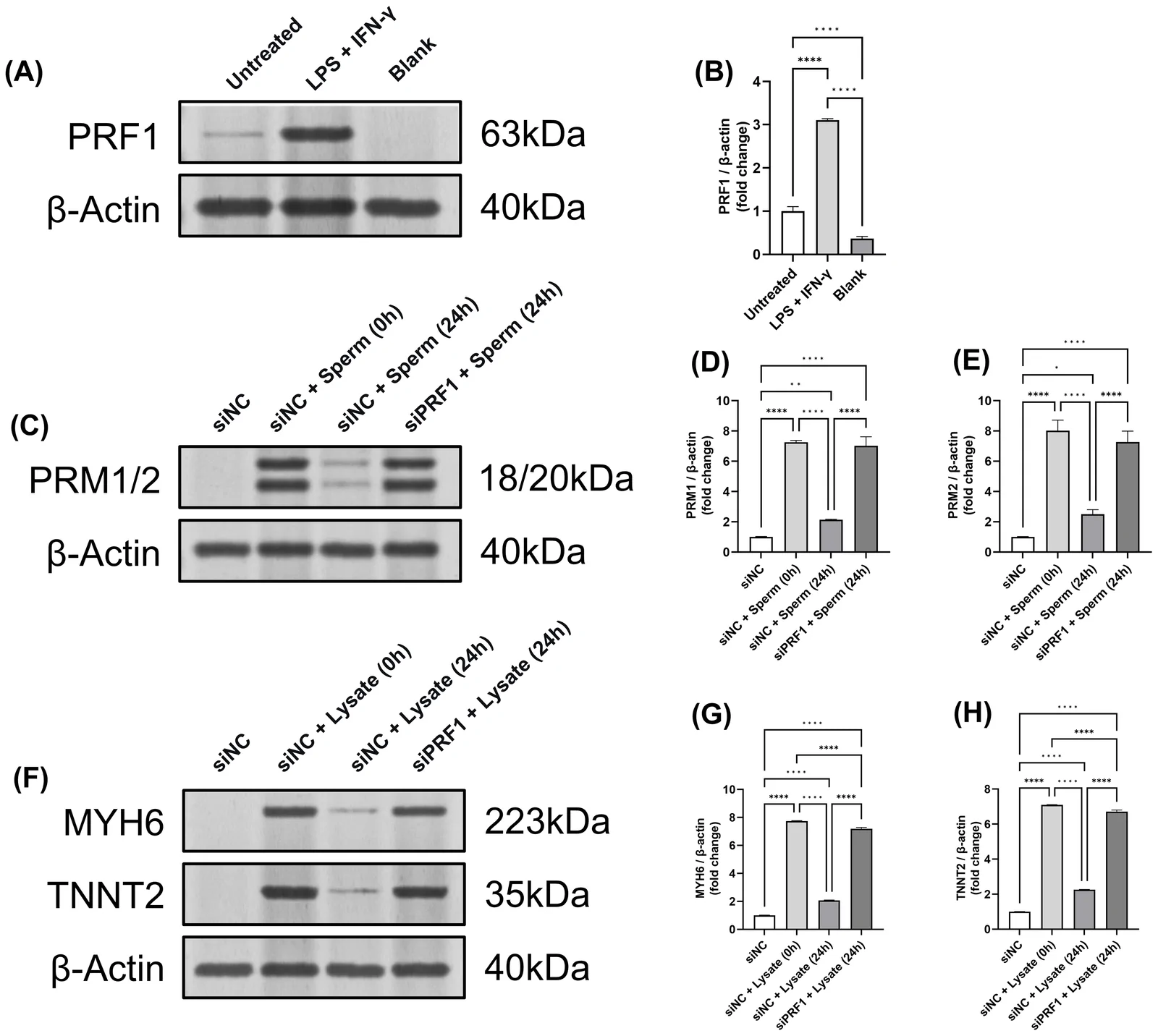
Western blot analysis of *PRF1* expression and antigen degradation. **(A)** Representative blots of *PRF1* protein expression; **(B)** Relative expression levels of *PRF1*; **(C)** Representative blots of *PRM1* and *PRM2* protein expression; **(D)** Relative expression levels of *PRM1*; **(E)** Relative expression levels of *PRM2*; **(F)** Representative blots of *MYH6* and *TNNT2* protein expression; **(G)** Relative expression levels of *MYH6*; **(H)** Relative expression levels of *TNNT2*. Data are shown as mean ± SD (n = 3). *p < 0.05, **p < 0.01, ****p < 0.0001.

## 4 Discussion

In this study, *SLAMF7*, *IL2RA*, and *PRF1* were identified as a set of core genes co-expressed in macrophages from both cardiac and testicular tissues. Loss of any of these genes induced highly similar transcriptional responses regardless of tissue origin, characterized by consistent upregulation of *C1QA*, *C1QB*, *C1QC*, the scavenger receptors *STAB1* and *FOLR2*, the inflammatory signaling molecules *NFKBIA* and *IL1B*, the metabolic regulator *ACOD1*, the chemokine *CCL5*, and the NLRP3 inflammasome. This conserved transcriptional response points to a shared immunosuppressive function of these three genes across both tissues. Previous studies have established that complement activation and scavenger receptor upregulation drive myocardial fibrosis (21, 22), and that aberrant expression of C1q and scavenger receptors in testicular macrophages disrupts immune privilege and promotes anti-sperm immunity (23, 24). Our findings suggest that these previously separate pathological processes may share a common molecular basis, with HF and NOA representing distinct manifestations of the same regulatory program dysregulated in different tissue environments.

Within this conserved transcriptional response, the upregulation of *ACOD1* and *S100A8/S100A9* warrants particular attention. *ACOD1* encodes aconitate decarboxylase, the key enzyme that catalyzes itaconate production in macrophages. Itaconate exerts anti-inflammatory effects by alkylating *KEAP1* to activate *Nrf2* (25). In the heart, the *ACOD1*/itaconate pathway limits excessive inflammatory responses following myocardial injury by inhibiting succinate dehydrogenase (SDH), thereby interfering with oxidative phosphorylation and driving a metabolic shift toward aerobic glycolysis in macrophages (26). *S100A8* and *S100A9* form the calprotectin complex, which regulates mitochondrial function and oxidative stress in macrophages (27, 28). In the cardiac context, *S100A8*/*A9* has been identified as a prognostic biomarker for heart failure after acute myocardial infarction, and targeting this pathway has been shown to ameliorate heart failure with preserved ejection fraction (HFpEF) by modulating *TLR4*/*NF-κB*-mediated inflammation (29). In the testis, *S100A8*/*A9* is highly expressed in macrophages within the autoimmune orchitis model and is markedly elevated in the seminal plasma of patients with oligoasthenozoospermia (OA) (30), whereas the role of the *ACOD1*/itaconate pathway in the testis has not yet been reported. The transcriptional upregulation of *ACOD1* and *S100A8/S100A9* observed in our study suggests adaptive changes in the expression of metabolism-related genes in macrophages following loss of the core genes. These observations link the immunoregulatory functions of the core genes to known immunometabolic pathways and provide clues for understanding the potential role of immunometabolism in HF and NOA.

In the testis, large numbers of apoptotic sperm cells are cleared daily. Our in silico knockout simulations showed that *PRF1* deficiency led to ectopic expression of the protamine genes *PRM1*, *PRM2*, and *TNP1* in macrophages, along with significant upregulation of *IRF8* and *S100A8*/*S100A9*. These findings suggest a non-canonical role of *PRF1* in macrophages, involving the degradation of sperm chromatin within phagolysosomes. *IL2RA* deficiency overactivated the *IRF8*-*TLR2* axis and upregulated multiple testis-specific genes, whereas *SLAMF7* deficiency primarily enhanced MHC-II and interferon pathways. These three genes thus exhibit a division of labor. Failure of any one link may lead to persistent sperm self-antigens, which can activate T cells via MHC-II-dependent pathways and ultimately trigger anti-sperm immune attacks and spermatogenic failure. This mechanism is consistent with the known model of autoimmune orchitis (31). *PRF1* is classically expressed in the cytolytic granules of NK cells and cytotoxic T lymphocytes (32). However, recent studies have linked *PRF1* mutations to macrophage activation syndrome and demonstrated that perforin regulates macrophage-mediated inflammatory responses in a gout model (33, 34). These findings parallel the non-canonical role of *PRF1* in macrophages identified in our study.

In the heart, necrotic cardiomyocytes release self-antigens, which include myosin, cardiac troponin, and mitochondrial DNA. These antigens also require timely clearance by macrophages (35), particularly following myocardial infarction or during prolonged pressure overload (36). Previous studies have shown that impaired clearance of cardiac self-antigens is an important trigger of chronic inflammation and ventricular remodeling (37, 38). Our results showed that *SLAMF7* deficiency activated the prostaglandin pathway, as evidenced by upregulation of *HPGDS* and *PGD2*; *IL2RA* deficiency induced M2-like polarization, with increased *FOLR2* and *LYVE1*; and *PRF1* deficiency enhanced C1q expression. Although the specific mechanisms of these three inflammatory patterns differ, they collectively point to a loss of the macrophage’s ability to limit excessive inflammation. This functional impairment can be transmitted to target cells via paracrine signaling. Conditioned medium experiments showed that soluble factors secreted by these macrophages directly induce inflammatory responses and dysfunction in both cardiomyocytes and Sertoli cells. Notably, upregulation of C1q, NLRP3, and multiple scavenger receptors in HF macrophages was similar to that observed in NOA macrophages. These findings suggest that deficiency of *PRF1*, *IL2RA*, or *SLAMF7* similarly impairs cardiac self-antigen clearance and may contribute to a vicious cycle of chronic inflammation and fibrosis, further supported by pHrodo flow cytometry and Western blot analyses.

Transcription factor network analysis identified *NFKB1*, *RELA*, *STAT1*, *STAT3*, *STAT4*, *STAT5A*/*B*, *GATA3*, and *FOS* as upstream regulators of *SLAMF7*, *IL2RA*, and *PRF1*. The target genes of these transcription factors were enriched in complement activation, inflammasome regulation, macrophage polarization, and MHC-II antigen presentation, consistent with the macrophage phenotypes observed in our knockout simulations. Previous studies have shown that the *NF-κB* signaling pathway serves as a key switch for macrophage responses to damage-associated molecular patterns (39), and its persistent activation can lead to NLRP3 inflammasome-dependent pyroptosis and tissue fibrosis (40–42). *STAT* family members, particularly *STAT1* and *STAT3*, are known to regulate macrophage polarization, complement gene expression, and MHC-II transcription (43–45). Based on these findings, we propose that activation of these upstream transcription factors may contribute to the conserved transcriptional response in macrophages following loss of the core genes, and that these transcription factors may also respond to inflammatory signals in the microenvironment, thereby forming a positive feedback amplification loop.

This study has several potential translational implications. First, the combination of *SLAMF7*, *IL2RA*, and *PRF1* may serve as a candidate biomarker panel for shared disease screening, particularly in patients with unexplained cardiomyopathy accompanied by fertility disorders. Second, targeting this common node in macrophages may represent a shared target worthy of further exploration in future preclinical and clinical studies. Direct agonists for these genes are currently unavailable, and future development of engineered *IL-2* variants, agonistic anti-*SLAMF7* antibodies, or small-molecule *PRF1* activators is needed. Third, drugs targeting upstream transcription factors such as *STAT3* and *NF-κB* may indirectly suppress pro-inflammatory signals and restore macrophage homeostasis, providing a potential strategy for simultaneous intervention in both diseases.

We acknowledge that additional work is needed. First, WGCNA analysis included only positively correlated modules, and the potential protective mechanisms in negatively correlated modules warrant further investigation. Second, although the *in vitro* validation was performed in a mouse system while the core genes were identified from human transcriptomic data, these genes are highly conserved between human and mouse. Given that the study focuses on the fundamental macrophage function of antigen clearance, the mouse system still provides valuable information, though validation in human systems would further clarify its clinical relevance. In addition, while the conditioned medium experiments showed that soluble factors secreted by macrophages exert pro-inflammatory effects on target cells, whether these effects arise directly from cytokine signaling or secondarily from metabolic or mitochondrial dysfunction in target cells remains to be determined. Moreover, the *in vivo* functions of the core genes remain to be confirmed using conditional knockout animal models, and assessment of genetic variants and protein expression levels in HF and NOA patient cohorts would also facilitate clinical translation.

In summary, this study connects HF and NOA through an immune regulatory node in macrophages composed of *SLAMF7*, *IL2RA*, and *PRF1*. Dysfunction of this node is associated with a common complement-inflammation activated state in macrophages, whereas the final disease outcome is determined by the type of organ-specific self-antigen. These findings provide a unified molecular perspective for the shared management of both diseases and lay a foundation for developing therapeutic strategies that simultaneously target cardiovascular and reproductive system disorders.

## 5 Conclusion

This study identifies *SLAMF7*, *IL2RA*, and *PRF1* as common molecular nodes that connect HF and NOA. A logistic regression diagnostic model based on these three genes showed moderate discriminative performance in external validation. Mechanistically, all three genes perform conserved immune-negative regulatory functions in macrophages. Their loss cooperatively upregulates C1q, scavenger receptors, the NLRP3 inflammasome, and related inflammatory factors, thereby generating a cross-disease transcriptional signature. Despite this common transcriptional response, the pathological consequences of losing the same gene differ markedly between the testicular and cardiac microenvironments. In the testis, *PRF1* loss impairs sperm antigen clearance, *IL2RA* loss overactivates the *IRF8-TLR2* axis, and *SLAMF7* loss enhances MHC-II and interferon pathways. Their combined function maintains testicular immune privilege, whereas their failure can trigger anti-sperm immune attacks and spermatogenic failure. In the heart, *SLAMF7* loss activates the prostaglandin pathway, *IL2RA* loss induces M2-like polarization, and *PRF1* loss enhances complement activation while also directly impairing cardiac self-antigen clearance. The combined dysregulation of these three genes is associated with chronic inflammation and fibrosis. Upstream transcription factor network analysis further reveals that *NF-κB* and *STAT* family transcription factors regulate these core genes. Collectively, this study provides a unified immunological mechanism for HF and NOA. It also suggests that targeting this common node in macrophages may offer a potential therapeutic direction for future investigation.

## Data Availability

The datasets presented in this study can be found in online repositories. The names of the repository/repositories and accession number(s) can be found below: https://www.ncbi.nlm.nih.gov/geo/, GSE5406, GSE57338, GSE45885, GSE45887, GSE25518, GSE183852, GSE149512.
