# Supplementary material for: Impaired antigen clearance of macrophages associates with heart failure and non-obstructive azoospermia: insights from transcriptomics, in silico knockout, and in vitro validation
Source: Front Immunol. 2026 Sep 15;17:1909437. doi: 10.3389/fimmu.2026.1909437 (PMC13619400; doi:10.3389/fimmu.2026.1909437)
Supplement: Supplementary file 1 [file DataSheet2.docx]

**Impaired Antigen Clearance of Macrophages Associates with Heart Failure and Non-Obstructive Azoospermia: Insights from Transcriptomics, In Silico Knockout, and In Vitro Validation**

**Supplementary Materials**

Figure S1. WGCNA network construction and module validation. (A) Gene clustering dendrogram for the HF dataset. (B) Soft‑thresholding power selection plot for the HF dataset. (C) Scatter plot of module membership (MM) versus gene significance (GS) for ME13 in the HF dataset. (D) Gene clustering dendrogram for the NOA dataset. (E) Soft‑thresholding power selection plot for the NOA dataset. (F) Scatter plot of module membership (MM) versus gene significance (GS) for ME1 in the NOA dataset.

Figure S2. pHrodo-based flow cytometry analysis of antigen degradation in macrophages. (A) Representative histograms of pHrodo fluorescence intensity in each treatment group under sperm co-culture. (B) Representative histograms of pHrodo fluorescence intensity in each treatment group under cardiomyocyte lysate stimulation.

Table S1. Top 15 enriched GO-BP terms in the HF ME13 module

| GO ID | Description | GeneRatio | BgRatio | RichFactor | p.adjust | Count |
| --- | --- | --- | --- | --- | --- | --- |
| GO:0030198 | extracellular matrix organization | 18/223 | 331/18860 | 0.05438 | 9.39e-05 | 18 |
| GO:0043062 | extracellular structure organization | 18/223 | 332/18860 | 0.05422 | 9.39e-05 | 18 |
| GO:0045229 | external encapsulating structure organization | 18/223 | 333/18860 | 0.05405 | 9.39e-05 | 18 |
| GO:0031589 | cell-substrate adhesion | 17/223 | 369/18860 | 0.04607 | 0.00148 | 17 |
| GO:0007566 | embryo implantation | 7/223 | 71/18860 | 0.09859 | 0.01011 | 7 |
| GO:0030199 | collagen fibril organization | 7/223 | 71/18860 | 0.09859 | 0.01011 | 7 |
| GO:0071559 | response to transforming growth factor beta | 14/223 | 318/18860 | 0.04403 | 0.01117 | 14 |
| GO:0141091 | TGF‑beta receptor superfamily signaling pathway | 16/223 | 411/18860 | 0.03893 | 0.01117 | 16 |
| GO:0003018 | vascular process in circulatory system | 13/223 | 283/18860 | 0.04594 | 0.01117 | 13 |
| GO:0060348 | bone development | 11/223 | 220/18860 | 0.05000 | 0.01840 | 11 |
| GO:0003012 | muscle system process | 17/223 | 485/18860 | 0.03505 | 0.01840 | 17 |
| GO:0007178 | cell surface receptor protein serine/threonine kinase signaling | 16/223 | 446/18860 | 0.03587 | 0.02023 | 16 |
| GO:0071560 | cellular response to TGF‑beta stimulus | 13/223 | 311/18860 | 0.04180 | 0.02023 | 13 |
| GO:0001894 | tissue homeostasis | 12/223 | 275/18860 | 0.04364 | 0.02188 | 12 |
| GO:0060249 | anatomical structure homeostasis | 12/223 | 275/18860 | 0.04364 | 0.02188 | 12 |

Notes: This table displays only the top 15 most significant GO-BP terms (ranked by ascending FDR) in the HF ME13 module. The complete results are provided in the Supplementary Data file.

Table S2. Top 15 enriched GO-BP terms in the NOA ME1 module

| GO ID | Description | GeneRatio | BgRatio | RichFactor | p.adjust | Count |
| --- | --- | --- | --- | --- | --- | --- |
| GO:0071706 | tumor necrosis factor superfamily cytokine production | 53/2246 | 201/18860 | 0.26368 | 2.24e-05 | 53 |
| GO:1903555 | regulation of TNF superfamily cytokine production | 53/2246 | 201/18860 | 0.26368 | 2.24e-05 | 53 |
| GO:0032640 | tumor necrosis factor production | 52/2246 | 196/18860 | 0.26531 | 2.24e-05 | 52 |
| GO:0032680 | regulation of tumor necrosis factor production | 52/2246 | 196/18860 | 0.26531 | 2.24e-05 | 52 |
| GO:1903557 | positive regulation of TNF superfamily cytokine production | 34/2246 | 117/18860 | 0.29060 | 0.00055 | 34 |
| GO:0032760 | positive regulation of tumor necrosis factor production | 33/2246 | 113/18860 | 0.29204 | 0.00059 | 33 |
| GO:0050867 | positive regulation of cell activation | 81/2246 | 401/18860 | 0.20200 | 0.00093 | 81 |
| GO:0071216 | cellular response to biotic stimulus | 60/2246 | 274/18860 | 0.21898 | 0.00122 | 60 |
| GO:0002696 | positive regulation of leukocyte activation | 77/2246 | 381/18860 | 0.20210 | 0.00122 | 77 |
| GO:0002367 | cytokine production involved in immune response | 35/2246 | 130/18860 | 0.26923 | 0.00122 | 35 |
| GO:0002718 | regulation of cytokine production in immune response | 35/2246 | 130/18860 | 0.26923 | 0.00122 | 35 |
| GO:0031666 | positive regulation of lipopolysaccharide‑mediated signaling | 8/2246 | 11/18860 | 0.72727 | 0.00236 | 8 |
| GO:0009636 | response to toxic substance | 58/2246 | 270/18860 | 0.21481 | 0.00236 | 58 |
| GO:0070665 | positive regulation of leukocyte proliferation | 41/2246 | 173/18860 | 0.23699 | 0.00452 | 41 |
| GO:0071219 | cellular response to molecule of bacterial origin | 53/2246 | 247/18860 | 0.21457 | 0.00530 | 53 |

Notes: This table displays only the top 15 most significant GO-BP terms (ranked by ascending FDR) in the NOA ME1 module. The complete results are provided in the Supplementary Data file.

Table S3. Shared Immune‑Related Candidate Genes in Heart Failure and Non-Obstructive Azoospermia

Gene Symbol

CD3E

CD3G

CD247

CD27

CD80

CD83

CD96

CLEC4E

CLDN1

CIITA

CPA4

CST7

CYP7B1

FLRT3

GZMK

GPR171

H3C12

IKZF3

IL2RA

IRAG2

KLRB1

LY9

NRIP3

OGFRL1

P2RY10

PLEK2

PRF1

SLAMF7

SKAP1

ST8SIA4

TNFSF8

TNFSF15

CD40LG

DRC11

BNC1

Table S4. Selection Frequency of Candidate Genes by Machine Learning Algorithms

| Gene Symbol | HF (count) | NOA (count) |
| --- | --- | --- |
| PRF1 | 5 | 5 |
| IL2RA | 5 | 5 |
| SLAMF7 | 5 | 4 |
| CD3G | 3 | 4 |
| CD3E | 3 | 4 |
| KLRB1 | 3 | 4 |
| GZMK | 5 | 1 |
| CD247 | 4 | 2 |
| CD40LG | 1 | 3 |
| CD96 | 3 | 1 |
| CD80 | 0 | 2 |
| CD27 | 0 | 2 |

Table S5. Average Expression of the Core Genes Across Cell Subpopulations in HF (log1p normalized)

| Cell Subpopulation | SLAMF7 | IL2RA | PRF1 |
| --- | --- | --- | --- |
| Fibroblasts | 0.0016 (0.1%) | 0.0018 (0.2%) | 0.0429 (0.7%) |
| Smooth muscle | 0.0150 (0.8%) | 0.0022 (0.1%) | 0.1444 (2.0%) |
| Pericytes | 0.0033 (0.2%) | 0.0025 (0.2%) | 0.0801 (1.0%) |
| Endothelial | 0.0017 (0.1%) | 0.0016 (0.2%) | 0.0249 (0.4%) |
| Macrophages | 0.0850 (5.5%) | 0.1304 (7.8%) | 0.0085 (0.3%) |
| T cells | 0.1231 (10.1%) | 0.0473 (3.1%) | 1.8936 (53.2%) |
| Cardiomyocytes | 0.1019 (8.3%) | 0.0318 (2.5%) | 2.2229 (33.8%) |

Notes： Values in parentheses indicate the percentage of cells expressing the gene within the subpopulation.

Table S6. Average Expression of the Core Genes Across Cell Subpopulations in NOA (log1p normalized)

| Cell Subpopulation | SLAMF7 | IL2RA | PRF1 |
| --- | --- | --- | --- |
| Fibroblasts | 0.0015 (0.1%) | 0.0006 (0.0%) | 0.0017 (0.1%) |
| Sertoli | 0.0013 (0.1%) | 0.0001 (0.0%) | 0.0028 (0.2%) |
| Macrophages | 0.0180 (1.3%) | 0.0425 (2.9%) | 0.0006 (0.1%) |
| Leydig | 0.0062 (0.3%) | 0.0029 (0.2%) | 0.0007 (0.1%) |
| Spermatocytes | 0.0155 (0.9%) | 0.0005 (0.0%) | 0.0121 (0.6%) |
| Spermatids | 0.0011 (0.1%) | 0.0008 (0.1%) | 0.0019 (0.1%) |
| Endothelial | 0.0063 (0.4%) | 0.0000 (0.0%) | 0.0060 (0.3%) |
| T cells | 0.2051 (13.8%) | 0.0180 (1.1%) | 0.5011 (31.1%) |
| Spermatogonia | 0.0000 (0.0%) | 0.0028 (0.3%) | 0.0008 (0.0%) |

Notes：Values in parentheses indicate the percentage of cells expressing the gene within the subpopulation.

Table S7. Key Transcription Factors Regulating the Core Genes

| TF | Target Gene(s) | Mode | HF log₂FC (FDR) | NOA log₂FC (FDR) | HF Activity (FDR) | NOA Activity (FDR) | Correlation with Score (HF) | Correlation with Score (NOA) |
| --- | --- | --- | --- | --- | --- | --- | --- | --- |
| FOS | IL2RA | Activating | -0.093 (1.000) | -1.303 (0.005) | 0.190 | 0.043 | 0.124 | 0.216 |
| GATA3 | IL2RA | Activating | 0.006 (1.000) | 0.257 (1.000) | 0.696 | 0.005 | 0.098 | 0.378 |
| NFKB1 | IL2RA | Activating | 0.012 (1.000) | 0.164 (1.000) | 0.019 | 0.121 | 0.087 | 0.816 |
| NFKB2 | IL2RA | Repressing | -0.062 (1.000) | -0.408 (1.000) | <0.001 | 0.163 | 0.077 | 0.059 |
| REL | IL2RA | Activating | 0.004 (1.000) | 0.420 (0.633) | 0.108 | 0.243 | 0.454 | 0.383 |
| RELA | IL2RA | Activating | -0.162 (<0.001) | -0.181 (1.000) | <0.001 | 0.178 | 0.138 | 0.400 |
| STAT1 | IL2RA, PRF1 | Activating | 0.216 (<0.001) | 0.159 (1.000) | 0.460 | 0.003 | 0.076 | 0.001 |
| STAT3 | IL2RA, PRF1 | Activating | -0.525 (<0.001) | -0.026 (1.000) | <0.001 | 0.579 | 0.005 | 0.675 |
| STAT4 | IL2RA, PRF1 | Activating | 0.621 (<0.001) | -1.444 (0.680) | 0.460 | 0.417 | 0.203 | -0.670 |
| STAT5A | IL2RA, PRF1 | Activating | -0.026 (1.000) | 0.027 (1.000) | <0.001 | 0.669 | 0.199 | 0.663 |
| STAT5B | IL2RA, PRF1 | Activating | 0.012 (1.000) | -0.350 (1.000) | <0.001 | 0.719 | 0.099 | 0.288 |

Table S8. Sequences of primers for qRT-PCR.

| Gene | Forward primer (5′→3′) | Reverse primer (5′→3′) |
| --- | --- | --- |
| C1qa | GTGGCTGAAGATGTCTGCCGAG | TTAAAACCTCGGATACCAGTCCG |
| Folr2 | CCAGCAAGTGGACCAGAGTT | CAGTCCCAGCCTTTATGCCA |
| Il1b | TGGACCTTCCAGGATGAGGACA | GTTCATCTCGGAGCCTGTAGTG |
| Acod1 | GGTTTTCTCCAGTGCCCATA | CAACTTGCCAAGCTTCAACA |
| Hpgds | CCTGGGCAGACTTCTACTGG | AAACTGCAACACCCCTTGAG |
| Ccl5 | TGCTGCTTTGCCTACCTCTC | TTGGCACACACTTGGCGGTT |
| Nlrp3 | ATTACCCGCCCGAGAAAGG | TCGCAGCAAAGATCCACACAG |
| Prm2 | CGCTACCGAATGAGGAGCCCCAGTG | TTAGTGATGGTGCCTCCTACATTTCC |
| Irf8 | CTACCTGACAGCAGGGTGGT | GGCATACAGCTGCTCTACCTG |
| S100a8 | TGTCCTCAGTTTGTGCAGAATATAAA | TCACCATCGCAAGGAACTCC |
| Myh6 | GCAGAACAGTAAAATTGAGGACG | CGCAGCTTCTCCACCTTAG |
| Tnnt2 | CAGAGGAGGCCAACGTAGAAG | CTCCATCGGGGATCTTGGGT |
| Il6 | ACCAGAGGAAATTTTCAATAGGC | TGATGCACTTGCAGAAAACA |
| Tnfa | CCACCACGCTCTTCTGTCTA | AGGGTCTGGGCCATAGAACT |
| Ccl2 | AGGTCCCTGTCATGCTTCTG | AAGGCATCACAGTCCGAGTC |
| Gata4 | GCCTCTATCACAAGATGAACGGC | TACAGGCTCACCCTCGGCATTA |
| Wt1 | GAGAGCCAGCCTACCATCC | GGGTCCTCGTGTTTGAAGGAA |
| Tjp1 | GTTGGTACGGTGCCCTGAAAGA | GCTGACAGGTAGGACAGACGAT |
